# Molecular detection of *Leptospira* spp. from canine kidney tissues and its association with renal lesions

**DOI:** 10.14202/vetworld.2018.530-534

**Published:** 2018-04-25

**Authors:** Biswajit R. Dash, Vitthal S. Dhaygude, Prashant D. Gadhave, Kaustubh V. Garud, Dattatarya P. Kadam

**Affiliations:** Department of Veterinary Pathology, Bombay Veterinary College, Parel, Mumbai - 400 012, Maharashtra, India

**Keywords:** histopathology, kidneys, *Leptospira* spp, nephritis

## Abstract

**Aim:**

The study aimed to detect the prevalence of *Leptospira* spp. in kidney tissues collected during necropsy and to establish its association with renal lesions in dogs of Mumbai region.

**Materials and Methods:**

Kidney tissues from 40 dogs were collected during necropsy after gross examination and then fixed in neutral buffered formalin and Bouin’s fluid for histopathology and histochemistry, respectively. Kidney tissues were also collected for the detection of *Leptospira* spp. by polymerase chain reaction (PCR) in a sterile container and stored at −80°C until further processing.

**Results:**

Of 40 cases studied, 13 (32.5%) cases showed lesions of nephritis of varying histotype and severity. Glomerulonephritis was reported as the most common type of nephritis in 9 (69.23%) cases, and interstitial nephritis was recorded in 4 (30.76%) cases. Chronic and acute interstitial nephritis was observed in two cases each. Renal failure as a cause of death was found in 7 (17.5%) dogs. Of a total of 40 cases, 9 were found positive for pathogenic *Leptospira* spp. genome by PCR. However, of nine PCR-positive cases, only four cases showed lesions in kidneys as glomerulonephritis and interstitial nephritis in two cases each. The rest five cases positive for *Leptospira* spp. by PCR did not show any appreciable lesions in the kidneys.

**Conclusion:**

Leptospiral DNA was detected in 9 (22.5%) cases by PCR. Of these nine cases, only four cases showed renal lesions. Other five cases which were positive for *Leptospira* spp. by PCR did not show any appreciable gross and microscopic lesions in the kidneys which might be carriers for *Leptospira* spp. Considering variable reports on types of nephritis in *Leptospira* spp. infection and also the prevalence of non-pathogenic *Leptospira* spp., it is important to conduct an extensive study on the prevalence of *Leptospira* spp. and its association with renal lesions involving batteries of tests.

## Introduction

Kidney diseases are important clinical problems encountered in dogs and are of the frequent cause of illness and death. Nephrotoxins, infections, urinary crystals and calculi, neoplasms, ischemia, autoimmune diseases, and genetic/familial determinants can result in renal disease-causing nephritis, nephrosis, amyloidosis, renal pelvis disorders, and developmental abnormalities ultimately leading to renal failure [1].

Leptospirosis is a zoonotic disease caused by *Leptospira* genus affecting virtually all mammals and has a broad range of effects from mild clinical infection to multiple organ failures and ultimately death. In the recent past, the incidence of leptospirosis by various methods for the diagnosis of *Leptospira* infection has been reported high by various authors [2-4].

Renal involvement is common in leptospirosis. Scanty literature is available on the prevalence of leptospirosis in canines and its association with renal lesions in Mumbai region. This study was designed to detect *Leptospira* spp. in canine kidney samples and its association with renal lesions.

## Materials and Methods

### Ethical approval

The study was conducted on the dead carcasses of canines brought for post-mortem examination to the Department of Veterinary Pathology, BVC, Mumbai, hence approval from IAEC was not necessary.

### Collection of samples

A total of 40 dogs died at BSPCA hospital, and Teaching Veterinary Clinical Complex, Bombay Veterinary College, Parel, Mumbai, or presented by owners for postmortem examination were included in the study. The dogs were necropsied following the standard necropsy procedure. After thorough gross examination, pieces of kidneys demonstrating lesions were collected for microscopic examination in neutral buffered formalin and Bouin’s fluid for fixation. Some pieces of kidneys were also collected aseptically in a sterile container and then froze at −80°C for molecular work.

### Histopathology and histochemistry

For histopathological examination, the kidney tissues were processed by routine paraffin embedding method and stained by hematoxylin and eosin (H and E) stain [5]. After histopathological examination, kidney samples were further stained by various special stains such as the Congo red stain for amyloid [6], Masson’s trichrome stain for the connective tissue fibers [5,7], and periodic acid–Schiff (PAS) for the demonstration of glycogen [6].

### Molecular detection of Leptospiraspp.

The frozen kidney samples stored at −80°C were used for molecular detection of *Leptospira* spp. DNA from tissue samples was extracted using TRIZOL^®^ reagent [8]. The primers were designed to detect the SecY gene of pathogenic *Leptospira* spp. The amplification mixture contained 3 µl of the diluted template DNA, 12.5 µl of Master (Bioron GmbH^®^, Germany), 1 µl each of both the forward and reverse primers, 1 µl of green buffer, and 7.5 µl of NFW (Bioron GmbH^®^, Germany). The polymerase chain reaction (PCR) conditions for the round were as initial denaturation at 95°C for 5 min, followed by 40 cycles of denaturation at 94°C for 1 min, annealing at 57°C for 1 min, and extension at 72°C for 1 min; final extension was done at 72°C for 10 min. The resultant products obtained after PCR were subjected to electrophoresis on 1.8% agarose gel.

## Results

Of a total of 40 necropsied dogs, 19 cases revealed kidney lesions of varying type and severity. However, in only 7 cases (17.5%), kidney failure and associated uremia were diagnosed as a cause of death. Among different renal lesions, 13 (32.5%) cases revealed mild, moderate-to-severe nephritis; nephrosis was recorded in a total of 4 (10%) cases; 1 (2.5%) case showed lesions of amyloidosis, and 1 (2.5%) case was of the focal renal infarct. Renal neoplasms and other lesions were not observed in the study.

Among cases diagnosed as nephritis, 9 (69.23%) were classified as glomerulonephritis and 4 (30.76%) as interstitial nephritis. Tubular nephritis was not observed. Suppurative nephritis was also not observed in the present study. All nine cases diagnosed as glomerulonephritis were of a chronic progressive type. Interstitial nephritis was diagnosed in four cases. Two cases were of chronic interstitial nephritis, and two showed lesions of acute interstitial nephritis.

### Glomerulonephritis

**Figure-1:**
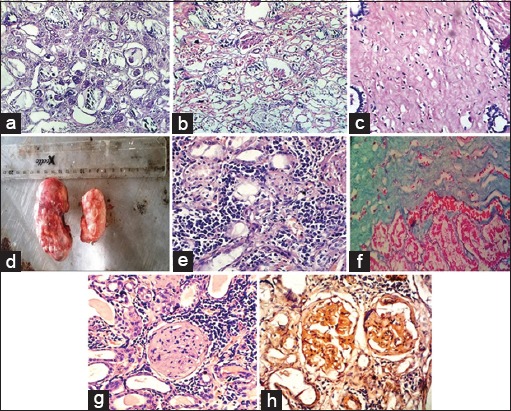
(a) Photograph showing severe glomerular atrophy (H and E, ×40). (b) Diffuse cystic dilation of tubules with necrotic changes (H and E, ×40). (c) Microphotograph showing collagen bundles and immature fibroblasts (H and E, ×40). (d) Gross picture depicting diffuse granular appearance of the kidneys. (e) Proliferation of fibrous connective tissue with infiltration of inflammatory cells (H and E, ×40). (f) Microphotograph showing collagen bundles and immature fibroblasts (40×, H and E Masson’s trichrome stain). (g) Pinkish homogenous deposits in glomerulus (H and E, ×40). (h) Microphotograph showing orange-to-red amyloid in glomerulus (×40 Congo red stain).

Histopathological examination revealed lesions predominantly in the glomerulus. However, lesions were also noted in tubules and interstitium. Glomerular lesions comprised glomerular atrophy (Figure-1a), hyalinization, and adhesions with basement membranes, increased cellularity, and deposition of pinkish material with varying severity and distribution in the majority of cases. In two cases, there was membranous glomerulonephritis characterized by proliferation of basement membranes, and five cases were diagnosed as proliferative glomerulonephritis and two as membranoproliferative glomerulonephritis. In few cases, necrosis was also evident. There was also the involvement of tubules and interstitium. Tubular lesions were comprised focal to multifocal degenerative and necrotic changes of varying severity. In few cases, there was focal to multifocal cystic dilatation (Figure-1b). Bluish-tinged necro-calcified mass in tubules was also observed in one case. Interstitial lesions were comprised focal, multifocal to diffuse infiltration of mononuclear cells, predominantly plasma cells, lymphocytes, and very few polymorphonuclear cells. The proliferation of fibrous connective tissue with thin-to-thick mature collagen bundles and immature fibroblasts in few cases was also noted with varying degrees of severity in either or both the kidneys (Figure-1c). For microscopic classification of lesions along with routine H and E stain, certain special stains, namely, Congo red to demonstrate amyloid, Masson’s trichrome to demonstrate collagen, and PAS to demonstrate basement membrane proliferation were also employed.

### Interstitial nephritis

Gross examination of two cases diagnosed as chronic interstitial nephritis revealed focal paleness and congestion, adhesions of the capsule with renal parenchyma, smooth appearance in one, diffuse granular in one case with varying severity, and distribution in either or both the kidneys (Figure-1d).

Histopathological examination revealed lesions predominantly in the interstitium. However, lesions were also noted in glomerulus and tubules in few cases. Lesions in interstitium were comprised focal, multifocal to diffuse proliferation of fibrous connective tissue. Infiltration of inflammatory cells predominantly of mononuclear cells was also recorded as the prominent lesion (Figure-1e). The section was simultaneously stained with Masson’s trichrome for collagen bundles and fibroblasts (Figure-1f). Tubular lesions were comprised focal to multifocal tubular necrosis and degeneration.

The other two cases were diagnosed as acute interstitial nephritis. Gross examination of the kidneys from these two cases revealed lesions, namely, swelling and dark reddish discoloration (congestion) in both the kidneys. The capsule was tense. The surface texture of the kidneys was smooth. White necrotic patches were also seen over the surface of the kidneys. Microscopic examination of the kidneys from the same cases showed lesions such as multifocal to diffuse severe infiltration of inflammatory cells mostly of mononuclear cells, plasma cells, and few neutrophils. However, there were no appreciable lesions in tubules and glomerulus.

One of the cases diagnosed as chronic progressive glomerulonephritis also revealed lesions of amyloidosis. Grossly, the affected kidneys showed changes such as paleness, capsule hard to peel off, and consistency focally firm and hard. A histopathological study by routine H and E stain revealed eosinophilic homogenous material deposited in the capillary loops of the glomerulus and the basement membrane (Figure-1g). The kidney tissue sections were further stained with Congo red stain to demonstrate amyloid which appeared as orange-red deposits around the capillary tufts and in the basement membrane (Figure-1h).

Of 40 cases, one case was diagnosed as an infarct. One kidney showed roughly triangular-\wedge-shaped whitish focal patch surrounded by the zone of inflammation on gross examination. The infarct was seen extending from the cortex up to the medulla and was roughly around 1.5 cm×1.2 cm×1.6 cm in dimensions. Microscopically, the kidneys revealed a large focal area of coagulative necrosis characterized by loss of tubular cell details and intense eosinophilia. Multifocal tubular degeneration was also noted. There were multifocal hemorrhages also. Minimal infiltration of inflammatory cells was also observed in the kidney section.

### Molecular detection of Leptospiraspp.

**Figure-2:**
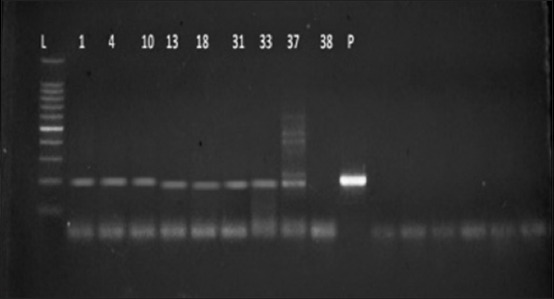
Detection of *Leptospira* spp. by polymerase chain reaction. Electrophoresis of the amplified products showing a product at 202 bp. Lane 1 (L): 100 bp DNA ladder, Lane 2-10: Kidney samples (1, 4, 10, 13, 18, 31, 33, 37, 38), Lane 11(P): Positive control, and Lane 12-17 (N): Negative controls.

A total of 40 kidney DNA tissue samples along with positive control, negative control, and 100 bp DNA ladder were subjected to electrophoresis. The expected product size was 202 bp, and a total of 9 (22.5%) kidney DNA samples were positive for *Leptospira* spp. (Figure-2). Of nine positive cases, only four cases revealed lesions and five did not show appreciable gross as well as microscopic lesions.

#### Gross findings in positive samples showing lesions

In most of the kidneys, the capsule was firmly adherent to the renal parenchyma at places and was hard to peel. In most of the kidneys, the consistencies of the kidneys were firm and hard with uneven surface contour due to fibrosis of the kidneys. Multifocal to diffuse patches of necrotic as well as hemorrhagic areas were observed on the surface of all the positive kidneys for leptospirosis.

#### Microscopic findings in positive samples showing lesions

On histopathological examination, two samples were diagnosed as glomerulonephritis and two cases as interstitial nephritis. The findings are as described in the previous chapter.

## Discussion

Many authors in the past have stated that death due to a renal failure associated with nephritis is highly prevalent in dogs [9-11] and glomerulonephritis was the most common and prevalent renal lesion seen in dogs [12,13]. We also in our study reported renal failure as a cause of death in 7 cases (17.5%) in dogs in Mumbai area. Nephritis (32.5%) was commonly seen and found to be the most common renal lesions in dogs, majorly glomerulonephritis (69.23%) [14]. Tubulointerstitial nephritis was widely prevalent in Chennai as stated by Di Bartola *et al*. [15].On the contrary, we found glomerulonephritis to be highly prevalent in dogs.

The seroprevalence for leptospirosis in dogs in Klang Valley, Malaysia, was 7.0%. Only one of the 19 dogs (5.3%) with kidney disease was tested positive for pathogenic *Leptospira* by PCR assay [16], which is in agreement with the current study.

The information on the exact incidence of amyloidosis is lacking, but several workers have regarded it as one of the uncommon lesions seen in kidneys [15,17-19]. In the present study, only 1 (2.5%) case of 40 cases revealed amyloidosis by a demonstration by the special stain. Hence, it can be regarded as important renal lesions, and studies involving large sample size should be conducted to know its exact prevalence. Renal amyloidosis is caused by varied etiological factors which lead to antigenic stimulation such as chronic infection, chronic inflammation, and neoplasia. Furthermore, familial nature of the disease has been reported. It is also caused by monoclonal B cell proliferation and dyscrasia of plasma cells and in some autoimmune diseases as well [19].

In India, epidemiological studies concluded that the prevalence of leptospirosis is high among canine patients by various diagnostic methods for detection of leptospiral antibodies as well as antigen in some studies [2,3]. In the present study also, the high prevalence of leptospiral DNA was reported in kidney tissues.

200 different serovars have been identified in the *Leptospira interrogans* complex [20]. Serovars of *L. interrogans* thought to be pathogenic to dogs are *icterohaemorrhagiae, canicola, pomona, grippotyphosa*, and *autumnalis* that are prevalent in India [21].

In this study, four samples positive for *Leptospira* spp. infection showed lesions in the kidneys. The gross findings were variable from normal reddish-brown kidneys to diffusely congested and from normal structure of kidneys to highly fibrotic and shrunken. On histopathology, two tissue samples were diagnosed as glomerulonephritis with glomerular atrophy and interstitial fibrosis along with infiltration of chronic inflammatory cells. In the other sample, there was hyalinization of the glomerulus and infiltration of the chronic inflammatory cells in the interstitium. The other two samples were classified as chronic interstitial nephritis with minimal glomerular involvement and infiltration of cells such as fibroblasts, mononuclear cells, and plasma cells.

Similar findings were reported by Ortega-Pacheco *et al*. [22] in which renal lesions associated with positive titers against *Leptospira* spp. were mesangial proliferative glomerulonephritis (MPGN), MPGN and interstitial nephritis (MPGN+IN), nephrosclerosis, mesangial glomerulonephritis, and interstitial nephritis. McIntyre and Montogomery [23] studied gross and histopathological changes in the kidneys of dogs suffering from leptospirosis and reported interstitial nephritis in the acute stages characterized by cellular infiltration followed by scarring and fibrosis in later stages. In the most severe cases, the cellular infiltration was localized predominantly to the corticomedullary region. Rest of the five cases positive for *Leptospira* spp. did not reveal any lesion in the kidney in the present study. This means that these animals might be carriers of leptospiral infection without showing any clinical signs and subsequent changes in the histological structure of the kidneys [24-29]. Furthermore, animals recovering from leptospirosis may become asymptomatic carriers harboring virulent leptospires in the renal tubules and shedding infectious leptospires into the environment for prolonged periods [20,24,30].

## Conclusion

Kidneys were found to be affected in 19 of 40 dogs necropsied during the experimental period. Kidney failure and associated uremia were diagnosed as a cause of death in 7 (17.5%) cases. Nephritis was found to be the most common kidney lesion. The incidence of nephritis was 32.5% which can be regarded as high. Renal failure was also found to be the most common among different organ failures causing death in dogs. Chronic progressive glomerulonephritis was reported as the most common type of nephritis in dogs, followed by interstitial nephritis and no cases of tubulonephritis. Among nine *Leptospira*-positive cases, four showed lesions of nephritis. Glomerulonephritis was observed in two cases, and interstitial nephritis was observed in two cases of total four positive cases. Kidney tissues from remaining five *Leptospira*-positive cases did not show appreciable lesions. This indicates that PCR targeting secY gene might have amplified *Leptospires* in the kidney tissue of carrier hosts without any signs of clinical disease.

## Author’s Contributions

BRD and VSD planned and conducted the research work and prepared the manuscript. PDG and DPK contributed to histopathological examination of slides. KVG helped in getting cases and contributed in several aspects of research work. All authors read and approved the final manuscript.
